# Complete Genome Sequence of a Multidrug-Resistant, *bla*_NDM-1_-Expressing *Klebsiella pneumoniae* K66-45 Clinical Isolate from Norway

**DOI:** 10.1128/genomeA.00601-17

**Published:** 2017-07-06

**Authors:** Adam Heikal, Ørjan Samuelsen, Tom Kristensen, Ole Andreas Økstad

**Affiliations:** a Centre for Integrative Microbial Evolution (CIME), Faculty of Mathematics and Natural Sciences, University of Oslo, Blindern, Oslo, Norway; b Laboratory for Microbial Dynamics (LaMDa), Section for Pharmaceutical Biosciences, School of Pharmacy, University of Oslo, Blindern, Oslo, Norway; c Reference Centre for Detection of Antimicrobial Resistance, Department of Microbiology and Infection Control, University Hospital of North Norway, Tromsø, Norway; d Department of Pharmacy, Faculty of Health Sciences, UiT–The Arctic University of Norway, Tromsø, Norway; e Department of Biosciences, University of Oslo, Blindern, Oslo, Norway

## Abstract

Multidrug-resistant *Klebsiella pneumoniae* is a major cause of hospital-acquired infections. Here, we report the complete genome sequence of the multidrug-resistant, *bla*_NDM-1_-positive strain *K. pneumoniae* K66-45, isolated from a hospitalized Norwegian patient.

## GENOME ANNOUNCEMENT

Carbapenem-resistant *Enterobacteriaceae* (CRE), such as *Klebsiella pneumoniae*, are classified as critical on the World Health Organization’s list of priority pathogens (1). Resistance to carbapenems, the traditional last-resort treatment option for severe community- and hospital-acquired infections, has been reported at proportions up to 54% (2). While CRE remain rare in Scandinavia, their arrival and subsequent local expansion from high-prevalence locations, such as through the return of patients hospitalized overseas, is a concern (3, 4). In 2010, the carbapenem-resistant strain *Klebsiella pneumoniae* K66-45, belonging to sequence type 11 (part of CG258), was isolated and cultured from a catheter urine sample following the return of a Norwegian patient previously hospitalized in India (5). Antimicrobial susceptibility testing and PCR analysis revealed resistance to a wide range of antibiotics, including meropenem and ertapenem, and the presence (but not location) of *bla*_NDM-1_, which encodes the New Delhi metallo-β-lactamase-1 (NDM-1), as well as genes encoding several other β-lactamases (5). To facilitate further investigation into the genetic basis of CRE and the dissemination of carbapenemases among Gram-negative bacteria, the genome sequence of *K. pneumoniae* K66-45 was determined using next-generation sequencing technology.

Genomic DNA was prepared from an overnight culture of *K. pneumoniae* K66-45 using the MO BIO DNeasy UltraClean Microbial kit (Qiagen, USA). The DNA libraries for single-molecule real-time (SMRT) sequencing were constructed using the BluePippin preparative electrophoresis system (Sage Science, USA) with a 9-kb cutoff. Sequencing was performed using one SMRT cell of the Pacific Biosciences RS II instrument, using P6-C4 chemistry with a 360-min movie time. A total of 104,622 reads were assembled using HGAPv3 (SMRT Analysis version 2.3.0, Pacific Biosciences, USA). Artifact frameshifts in the PacBio sequence were corrected using Pilon (6), and Bowtie2 (7) was subsequently used to map 99.4% of available Illumina sequence reads (>300× coverage, GenBank accession no. NZ_LNGZ01000000) back to the Pilon-corrected PacBio consensus sequence. Annotation was performed with the NCBI Prokaryotic Genome Annotation Pipeline.

The *K. pneumoniae* K66-45 genome contains a single ~5.4-Mb chromosome, encoding the SHV-11 β-lactamase, and four plasmids, pK66-45-1 to pK66-45-4, which encode the multidrug resistance basis of the strain. The plasmid-based resistance determinants include *bla*_NDM-1_, *bla*_CTX-M-15_, *armA*, *aadA2*,* qnrS*, *dfrA12*, and *sul1* identified on pK66-45-1 (338,512 bp); *bla*_OXA-1_, *aac(6′)*-*Ib-cr*, and *aph(3′)-Ia* on pK66-45-2 (200,356 bp); and *bla*_OXA-1_, *bla*_CTX-M-15_, *bla*_OXA-9_, *bla*_TEM-1A_, *aac(6′)-Ib-cr*,* aac(6′)-Ib*, and* aadA1* on pK66-45-3 (120,533 bp). No resistance determinants were identified on pK66-45-4 (41,111 bp). As shown previously, the bleomycin resistance gene *ble*_MBL_ was located downstream of *bla*_NDM-1_ (8). This repertoire of genes illustrates the immense reservoir of resistance determinants associated with mobile genetic elements such as plasmids. The acquisition of these plasmids by pathogens such as *K. pneumoniae* is a major driver in the increase in multidrug resistance that now threatens modern medicine.

As CRE isolates remain rare in Scandinavia, this complete genome sequence will provide a valuable reference for investigations into the dissemination and evolution of multidrug resistance, as well as for improved infection control measures for CRE.

### Accession number(s).

This complete genome project has been deposited at GenBank under the accession numbers CP020901 to CP020905.
